# Dexmedetomidine Versus Fentanyl in Intraoperative Neuromuscular Monitoring Using A Propofol-based Total Intravenous Anaesthesia Regimen in Spine Surgeries

**DOI:** 10.4274/TJAR.2024.241670

**Published:** 2024-10-30

**Authors:** Medha Bhardwaj, Vijay Mathur, Ravindra Singh Sisodia, Sunita Sharma, Akash Mishra

**Affiliations:** 1 Mahatma Gandhi Medical College & Hospital, Mahatma Gandhi University of Medical Sciences & Technology, Jaipur, Clinic of Neuroanaesthesia, Jaipur, India; 2 Mahatma Gandhi Medical College & Hospital, Mahatma Gandhi University of Medical Sciences & Technology, Jaipur, Clinic of Community Medicine, Division of Biostatistics, Jaipur, India

**Keywords:** Dexmedetomidine, evoked potentials, fentanyl, hemodynamics, propofol

## Abstract

**Objective:**

This prospective, double-blind, randomized study aimed to compare the effects of dexmedetomidine and fentanyl on the latency and amplitude of transcranial motor evoked potentials (TcMEPs) under propofol-based total intravenous anaesthesia (TIVA) in spine surgery. Secondarily, intraoperative hemodynamics, total propofol consumption, recovery profile, and surgical field quality were compared.

**Methods:**

TcMEP amplitude and latency recordings of bilateral abductor pollicis brevis and abductor hallucis muscles posted for elective lumbar spine surgery under TcMEP monitoring randomly divided into two study groups. Throughout the surgery, TIVA was administered using intravenous propofol (100-150 µg kg^-1 ^min^-1^) and dexmedetomidine (0.5-0.7 µg kg^-1 ^h^-1^) in group D and intravenous propofol (100-150 µg kg^-1 ^min^-1^) and fentanyl (1 µg kg^-1 ^h^-1^) in group F. TcMEPs were recorded at various time points during the surgery. Immediately after extubation recovery from anaesthesia was noted. Additionally, hemodynamic parameters, total propofol consumption, and surgical field quality were assessed.

**Results:**

Latency and amplitude were comparable between the groups. Time to extubation was significantly longer in group D, but the mean (standard deviation) duration of stay in recovery was shorter in group D [47.55 (7.51) 95% confidence interval (CI) (44.863-50.237)] (*P*=0.046). Total propofol consumption was reduced in group D [220 (38) 95% CI (206.402-233.598)] (*P*=0.025) and surgical field condition was better in group D.

**Conclusions:**

Dexmedetomidine and fentanyl do not have any effect on TcMEP amplitude and latency. However, dexmedetomidine provides the additional advantage of reduced total propofol consumption, shorter stay in recovery, and better surgical field quality.

**Main Points**

• Dexmedetomidine and fentanyl had no effect on the transcranial motor evoked potential amplitude and latency.

• Dexmedetomidine reduces total propofol consumption, provides a better quality of surgical field.

• Dexmedetomidine provides a shorter stay in recovery.

## Introduction

Currently, monitoring of transcranial motor evoked potential (TcMEP) intraoperatively is routinely performed and is regarded as a vital tool available to the surgical team that guides them in avoiding any motor tract injury during certain surgeries of the spine and cranium.^1^

The motor cortex is stimulated through the skull to produce compound muscle action potentials (CMAP), which are produced from peripheral muscles to maintain the motor pyramidal pathway intact. TcMEP has 91% sensitivity and 96% specificity, making it a gold standard modality.^2^

Intraoperatively, several factors influence CMAP apart from surgical manipulation like blood pressure, temperature, expired carbon dioxide partial pressure, and oxygen, so for optimal TcMEP recording, all the aforementioned factors should be optimized.^3^ Anaesthetic agents like muscle relaxants, are known to block signal transmission over the neuromuscular junction. Inhalational agents should be used at a low minimum alveolar concentration to suppress CMAP. Opioids have a minimal influence on CMAP.^3, 4^ Intravenous (IV) anaesthetics are known to suppress the TcMEPs less in comparison to inhalational agents.^5^

Most commonly, propofol-based total IV anaesthesia (TIVA) along with opioid is used during TcMEP monitoring, which is recommended as an ideal regime by the American Society of Neurophysiological Monitoring. Propofol is metabolized rapidly so its effect on motor evoked potential (MEPs) and sedation can be titrated quickly. However, higher doses are required for maintaining the surgical depth then it may depress the TcMEP readings.^3, 6^ Therefore adjuvants like an opioid or dexmedetomidine can be employed for maintaining the anaesthetic depth without affecting the MEP.^7^

Modified Delphi consensus recommendations support using the standard regime of TIVA along with an adjuvant like dexmedetomidine, ketamine, or lignocaine without any effect on TcMEP signals.^8^

Therefore, we aimed to evaluate and compare the effects of dexmedetomidine and fentanyl in intraoperative neurophysiological monitoring using a propofol-based TIVA regimen in spine surgery.

## Methods

### Study Design

Prospective, randomized, double-blind study was conducted in strict compliance with the principles of the Declaration of Helsinki. Informed written consent from the patients and institutional Ethics Committee of Mahatma Gandhi Medical College & Hospital, Mahatma Gandhi University of Medical Sciences & Technology, Jaipur (approval no.: MGMC&H/IEC/JPR/2022/1148, date: 22.09.2022) were obtained before the conduct of this study. Registration with the Clinical Trials Registry - India (CTRI/2022/12/048497) was also performed. The study was conducted over a span of 1 year in which all patients of either sex, aged 18 to 65 years, posted for elective spine surgery under transcranial MEP monitoring with a Medical Research Council Scale motor power ≥4/5 were included. Patients who refused to participate, were allergic to the study drugs, had impaired renal and hepatic function, or had any contraindications to TcMEP monitoring like pacemaker, vascular clips, epilepsy, intracranial electrodes, or cortical lesions with raised intracranial pressure were excluded.

### Sample Size Determination

Sample size determination was based on the efficacy of dexmedetomidine and fentanyl in terms of the ratio of complete response (defined as no change in amplitude or latency of TcMEP potentials). We selected a baseline ratio of 40% for complete responses based on a previous study.^1^ Sample size of 32 patients in each group was derived, where 80% power was present at an alpha 0.05 to detect a difference of 30% between the two groups in terms of the ratio of complete response. Considering a dropout rate of approximately 5%, we calculated that 30 patients would be appropriate.

### Randomization, Allocation, Blinding

**Figure 1:**
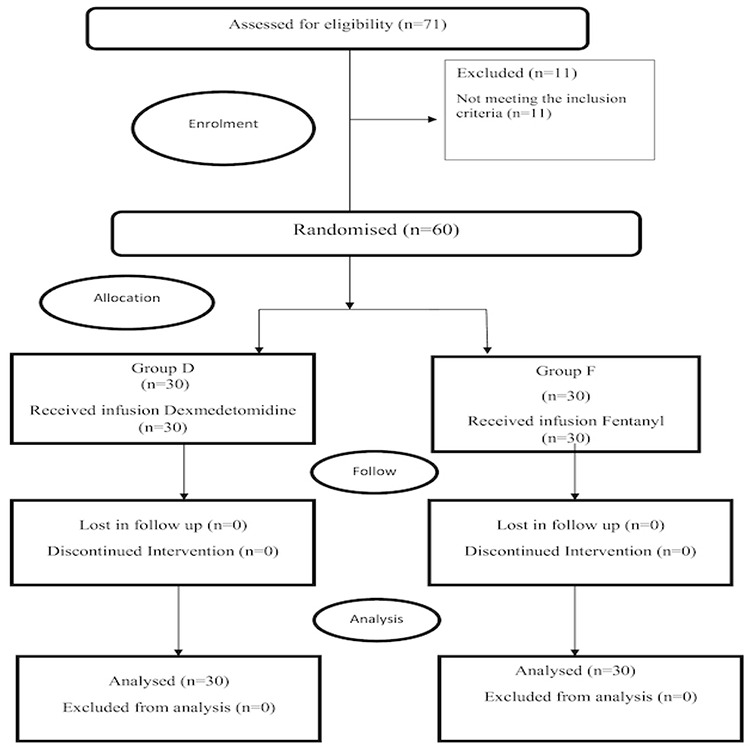
Consolidated standards of reporting trials (CONSORT) flow diagram of participants. D, dexmedetomidine; F, fentanyl; n, number of cases.

Sixty patients who fulfilled the inclusion criteria were distributed into two study groups with the help of a computer-generated random number table written in an opaque sealed envelope (Figure 1). For group D n = 30 patients, we administered infusion propofol [Neorof 10 mg mL^-1^ (20 mL), Neon laboratories limited, Mumbai, India] with infusion dexmedetomidine hydrochloride [Dexem 200 µg (2 mL), Themis medicare limited, Uttarakhand, India] prepared in a 50 ml syringe by adding normal saline (48 mL) making 4 µg mL^-1^ drug concentration.

Group F n = 30 patients received infusion propofol [Neorof 10 mg mL^-1^ (20 mL), Neon laboratories limited, Mumbai, India] along with infusion fentanyl citrate [Themifent 500 µg (10 mL), Themis medicare limited, Uttarakhand, India] prepared in a 50 mL syringe by adding normal saline (40 mL) making 10 µg mL^-1^ drug concentration.

An anaesthesiologist who is not associated with the study prepared all infusions. The patient and the anaesthesiologist administering the medications were unaware of the contents of the syringe.

A thorough pre-anaesthesia check-up was conducted where neuromonitoring was explained to the patients and consent was obtained. Any neurological deficit, including sphincter disturbance, was noted. Instructions were given to patients to remain nil oral for at least 6 hours (solid food) and 2 hours (clear liquids) before surgery. In operating theater, multipara monitor (MX-550 Philips Medizin Systeme, Germany) showing electrocardiogram, non-invasive BP monitoring, pulse oximetry, and temperature was attached. An IV access with a wide-bore cannula was secured. The anaesthesia regimen was standardized. Preoxygenation with 100% oxygen for at least 3 min, premedication with IV glycopyrrolate 4 µg kg^-1^ and IV fentanyl 2 µg kg^-1^. Induction was performed with IV propofol 2 mg kg^-1^, and once ventilation was confirmed, IV succinylcholine 2 mg kg^-1^ was administered to facilitate intubation. A bite block was placed to prevent tongue laceration. An arterial cannula was secured in the radial artery for monitoring beat-to-beat blood pressure. Neuromuscular blockade was monitored using a train-of-four (TOF) ratios in which electrodes were placed at the wrist for the ulnar nerve. Once the TOF ratio was >90%, baseline MEP readings were noted in supine position. Paracetamol 15-20 mg kg^-1^ IV was administered as an analgesic agent in both groups.

NIM-Eclipse (Medtronic, Minneapolis, MN, USA) was used to obtain MEP. Bispectral index (BIS) (Covidien Digital, MN, USA) monitoring was also used to guide the depth of anaesthesia. Using a skin probe, the temperature was recorded and maintained at 35-36 degrees Celsius using warming devices. Surgery was performed in the prone position. Hemodynamic variables like mean arterial pressure (MAP) and heart rate (HR), were documented every 30 min. For assessment of surgical field quality Former’s score was used, where 1- stands for only mild bleeding, with no surgical nuisance; 2- moderate bleeding, no surgical interference; 3- moderate bleeding, compromising field of surgery moderately; 4- heavy but controllable bleeding, significant interference with the surgery; and 5- for massive uncontrollable bleeding. scores of 1 and 2 were considered acceptable, whereas the rest were unacceptable.

Throughout the surgery, TIVA was administered using IV propofol (100-150 µg kg^-1 ^min^-1^) with dexmedetomidine (0.5-0.7 µg kg^-1 ^h^-1^) in group D whereas IV propofol (100-150 µg kg^-1 ^min^-1^) with fentanyl (1 µg kg^-1 ^h^-1^) in group F. The propofol and dexmedetomidine infusions were titrated to maintain BIS values between 40 and 60. Ventilator settings were adjusted to maintain end-tidal carbon dioxide within 35-45 mmHg. None of the neuromuscular blocking agents were used during surgery.

### TcMEP Recording

International 10-20 electrode placement system was used to place cork screws at C3 and C4. Six consecutive pulses with a duration of 0.5 ms were used for stimulation. A constant current with 70-200 mA strength at a time interval of 2-5 msec in between the two stimuli was applied. These settings were kept the same in all cases. Recordings from the upper limb were obtained from the abductor pollicis brevis muscle (C8, T1 median nerve innervation) that serves as the control, whereas the abductor hallucis muscle (L4, L5 medial plantar nerve) was used for the lower limbs. TcMEP were noted first in the supine position (Ts) as baseline, then after positioning the patient in prone (Tp), before any surgical manipulation (Tm), followed by subsequently as per the surgeon’s demand (Tm1, Tm2) and finally at completion of the surgery (Te).

All infusions were stopped prior to completion. The total requirement of propofol was also noted. The patient was turned to Ts and extubated. Immediately after extubation, the time to verbal response/eye opening (T1), time to extubation (T2), and duration of stay in recovery (T3) was noted.

Any untoward events, such as bradycardia, hypotension, tongue laceration, injury at the electrode insertion site, and any unwanted limb movements or respiratory efforts, were also recorded.

The MAP was maintained within 20% of the baseline in all cases. In case of a fall of MAP >20% of the baseline value, first, an IV fluid bolus was given with 200 mL but if there was persistent hypotension, then a mephenteramine 6 mg bolus IV was given. Any episode of hypertension (MAP>20% of baseline) was managed with IV Labetalol (5 mg) incrementally.

### Statistical Analysis

SPSS Statistics (IBM Corp. Released 2010. IBM SPSS Statistics for Windows, Version 23.0. The IBM Corp. (Armonk, NY: IBM Corp.) software was used for the analysis. All continuous variables are presented as mean ± standard deviation (SD) or median with interquartile range, depending on the normal condition of the data. The normalcy condition was checked using the Kolmogorov-Smirnov test before applying the parametric or non-parametric tests. Categorical data are presented as frequencies (percentage). The comparison of continuous variables like current mA, Latency, Amplitude, duration, age, height, weight, BIS, between the Dexmedetomidine and Fentanyl was done by using Independent Student’s t-test or Mann-Whitney U test depending upon the data distribution. Furthermore, the comparison of continuous variables within the groups at different time points was carried out using repeated measures ANOVA (RMANOVA) or Friedman’s test. All statistical tests were performed at a 5% significance level, and a *P* value of less than 0.05 was considered statistically significant.

## Results

### 1. Demographics

Demographic data were comparable between the two groups (Table 1).

### 2. TcMEP

No significant difference was found over time in latency and amplitude between the groups (Tables 2, 3).

Within dexmedetomidine, there was a decrease in the amplitude value in right upper limb (RUL) compared with baseline at Tp, Tm, and Tm1, and subsequent increase at Tm2 and Te was statistically as well as clinically insignificant (Table 3). In group D, latency decreased compared with baseline at all time intervals in RUL, which was clinically and statistically non-significant (Table 2).

Within the fentanyl group, latency was well preserved within the baseline value throughout the surgery in all four limbs, whereas there was an increase in the amplitude as compared with baseline in RUL at Tp, Tm, Tm1, and Te, which was statistically significant (Tables 2, 3).

### 3. Hemodynamics and BIS

MAP and HR were found to be comparable between both the groups. Although statistically non-significant, lower values were obtained in Group D than in Group F. Also lower BIS scores were recorded in group D compared with group F.

### 4. Recovery profile, complications, total propofol consumption, and former score

The time to response or eye opening was comparable. The time to extubation was significantly more in group D though statistically not significant but the mean (SD) duration of stay in recovery was 47.55 (7.51) [95% confidence interval (CI) (44.863-50.237)] in group D and 51.10 (8.73) [95% CI (47.976-54.224)] in group F, which was statistically significant (*P*=0.046) (Table 4).

Bradycardia was seen in 4 and 2 patients in groups D and F, respectively, which was statistically non-significant. Hypotension noted in 7 patients as compared to 3 in D group and F, respectively, which is statistically non-significant. None of the patients experienced tongue laceration or injury at the electrode site insertion (Table 4).

A statistically significant difference was noted in total propofol consumption, which was 220 (38) [95% CI (206.402-233.598)] in group D and 282 (140) [95% CI (231.903-332.097)] in F group (*P*=0.025) (Table 4).

**Figure 2:**
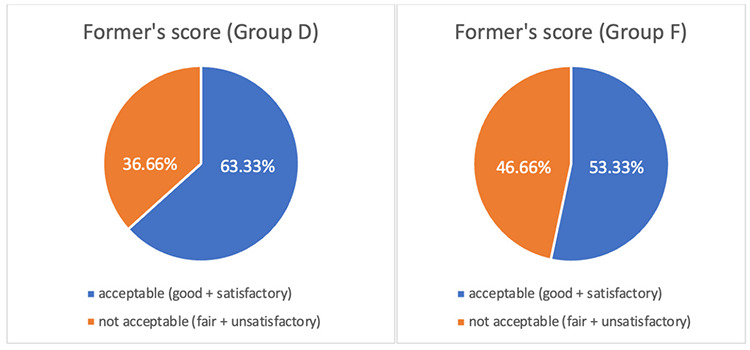
Former’s score in both the groups. D, dexmedetomidine; F, fentanyl.

Surgical field condition as determined using the Former’s score was better in group D than in group F, although statistically non-significant (*P*=0.436) (Figure 2).

## Discussion

While monitoring TcMEP, any interruption in the motor tract pathway is determined by either all or none phenomena (means whether there is generation of CMAP or not), or if there is >50% reduction in amplitude or an increase in latency by >10%.^9^ Recording of TcMEP might sound simple just like any other monitoring, but when it comes to practicality it requires expertise and advanced skills as a number of factors including anaesthetic agent affect both latency and amplitude.

We were able to successfully record TcMEP in all patients. Our primary objective was to note any change in latency and amplitude in both the upper and lower limb values between the dexmedetomidine and fentanyl groups at any given point in time, and we found no significant change in latency and amplitude in either group. This finding is consistent with previous studies in literature.^10, 11, 12^ However, there was a decrease in RUL amplitude compared with baseline at Tp, Tm, and Tm1 and a subsequent increase at Tm2 and Te, but these changes were statistically as well as clinically insignificant. This result could be attributed to the cumulative effect of loading doses of dexmedetomidine and propofol after induction.

Identical to our findings, various studies by Tobias et al.^12^, Tsaousi et al.^13^, Li et al.^14^, and Anschel et al.^15^ have reported no significant change in MEP latency or amplitude when using dexmedetomidine with propofol. Bala et al.^11^ found that dexmedetomidine until a plasma concentration of 0.6 ng mL^-1^ does not affect the MEP threshold current intensity and amplitude. All of these studies used the same dose of dexmedetomidine as used in our study.

We observed that there was a reduced consumption of propofol in group D, which is in agreement with a study on spinal surgeries by Tsaousi et al.^13^.

Our study showed that in group D, there was a significantly prolonged time to extubation compared with group F. This finding is contrary to most studies that showed no alteration in the recovery parameters whether dexmedetomidine was used alone or in combination with propofol.^14, 15, 16, 17, 18^ However, this can be explained by the fact that the elimination half-life of dexmedetomidine is 2-3 hours but the context-sensitive half-life is increased from 4 min after a 10 min continuous infusion to 250 min after an 8 h infusion.^19^ Hence, it may prolong recovery owing to analgesic and sedative actions and also a longer context-sensitive half-life in long-duration surgeries. However, there was a faster discharge from recovery in group D patients, which indicates overall better recovery.

Throughout the surgery at all points, the HR was lower in group D, although not statistically significant, which is in agreement with previous literature.^14, 15, 20, 21, 22, 23, 24^ We also report a statistically significant reduction in the total consumption of propofol as well as deepened plane of anaesthesia, as suggested by the lower BIS value in patients receiving dexmedetomidine. This finding is in agreement with the findings of a study by Panse et al.^1^ as well as in literature.^25^

To the best of our knowledge, no previous study has compared the surgical field quality during MEP recordings in spine surgeries, which makes our study unique. We found that dexmedetomidine provides better surgical field conditions, meaning that it helps maintain better hypotensive anaesthesia than fentanyl. This further provides an additional advantage of reduced bleeding from the surgical field. Our findings are consistent with those of Panse et al.^1^ where they used Former score to assess surgical field quality in surgeries for kyphoscoliosis correction but monitored only somatosensory-evoked potentials intraoperatively.

### Study Limitations

A few limitations are present in our study. Our sample size is relatively small. Plasma concentrations of the study drugs were not measured, so plasma concentrations can vary despite a fixed dose regime. Postoperative analgesic requirement was not studied. Further studies with larger sample sizes are needed to validate the findings of our study.

## Conclusion

Our findings revealed that better surgical field quality can be achieved using dexmedetomidine infusion with propofol-based TIVA. Both fentanyl and dexmedetomidine facilitate MEP recordings without any effect on amplitude or latency. Dexmedetomidine provides an additional advantage of reducing total propofol consumption and maintaining the depth of anaesthesia.

## Figures and Tables

**Table 1. Demographic Data of Patients in Groups D and F:** 

	**Group D (n = 30)**	**Group F (n = 30)**	***P* value**
Age (years)	43.30 (10.36)	41.73 (9.83)	0.551
Weight (kg)	61.27 (8.17)	62.23 (8.32)	0.652
Height (cm)	164.97 (7.79)	165 (7.80)	0.882
Gender (Male/Female)	20/10	21/9	0.677
ASA physical status (I/II)	24/6	25/5	0.334
The type of lumbar surgery Tumor (intradural extramedullary) Canal stenosis Listhesis Pott’s spine	18 05 05 02	17 03 09 01	0.819
Duration of surgery (min)	192 (21.71)	191.88 (20.14)	0.422

Data expressed as mean (standard deviation) or numbers.

ASA, American Society of Anesthesiologists; n, number of patients; D, dexmedetomidine; F, fentanyl; n, number of cases.

**Table 2. Comparison of Latency Between and Within Groups D and F:** 

**Time**	**Group D (n = 30)**	**Group F** **(n = 30)**		**Group D** **(n = 30)**	**Group F** **(n = 30)**		**Group D** **(n = 30)**	**Group F** **(n = 30)**		**Group D** **(n = 30)**	**Group F** **(n** *=* **30)**	
	**RUL**	**RUL**	***P*** **value**	**RLL**	**RLL**	***P*** **value**	**LUL**	**LUL**	***P*** **value**	**LLL**	**LLL**	***P*** **value**
Ts	29.40 (12.16)	29.31 (12.26)	0.97	26.23 (9.19)	27.94 (4.53)	0.38	31.31 (13.06)	25.86 (12.21)	0.51	25.79 (9.17)	27.26 (9.63)	0.54
Tp	27.82 (10.62)	26.98 (10.11)	0.75	26.93 (9.52)	28.61 (4.24)	0.38	27.79 (9.84)	30.23 (13.58)	0.42	25.27 (8.83)	26.14 (8.72)	0.70
Tm	26.82 (9.86)	26.55 (9.14)	0.91	26.29 (8.98)	27.78 (4.13)	0.41	28.37 (14.07)	26.14 (10.39)	0.48	25.11 (7.85)	26.90 (8.97)	0.41
Tm1	29.20 (11.62)	26.87 (10.19)	0.41	26.32 (9.01)	27.31 (4.35)	0.59	29.75 (10.02)	26.85 (9.59)	0.25	25.53 (8.44)	26.98 (8.42)	0.50
Tm2	27.71 (8.78)	27.48 (8.09)	0.92	26.32 (8.97)	26.92 (4.24)	0.74	29.45 (10.75)	27.65 (11.91)	0.54	25.74 (8.64)	27.19 (8.73)	0.52
Te	29.29 (11.21)	25.79 (9.19)	0.19	26.66 (4.80)	27.10 (4.09)	0.70	31.63 (11.10)	29.50 (12.73)	0.50	25.25 (8.78)	27.17 (8.79)	0.40
*P *value	0.635	0.335		0.718	0.432		0.662	0.442		0.487	0.053	

Data expressed as mean (standard deviation) or numbers. D, dexmedetomidine; F, fentanyl; n, number of cases; RUL, right upper limb; RLL, right lower limb; LUL, left upper limb; LLL, left lower limb; Ts, baseline latency in the supine position; Tp, latency in the prone position; Tm, latency before any manipulation; Tm1, latency as per the surgeon’s demand; Tm2, latency as per the surgeon’s demand; Te, latency at the end of surgery (Te).

**Table 3. Comparison of Amplitude Between and Within Groups D and F:** 

**Time**	**Group D** **(n = 30)**	**Group F** **(n = 30)**		**Group D** **(n = 30)**	**Group F** **(n = 30)**		**Group D** **(n = 30)**	**Group F** **(n = 30)**		**Group D** **(n = 30)**	**Group F** **(n = 30)**	
	**RUL**	**RUL**	***P*** **value**	**RLL**	**RLL**	***P*** **value**	**LUL**	**LUL**	***P*** **value**	**LLL**	**LLL**	***P*** **value**
Ts	172.40 (130.75,248.20)	180 (139.50,234.25)	0.900	194.50 (137,302)	152 (108,240.25)	0.204	203.05 (123.75,245.35)	139.95 (107.50,236.50)	0.438	224 (109,265.75)	223 (105.05,278.25)	0.767
Tp	170 (139.75,251)	186 (140.47,236)	0.988	222.50 (136.10,275.25)	157.50 (110.87,235.25)	0.143	210.50 (125,245)	141.50 (107.50,235.25)	0.371	174.50 (125.15,288.20)	163 (115.75,269.75)	0.446
Tm	166.45 (143.75,242.25)	184 (145.25,235.75)	0.751	187.30 (121.07,247.05)	176 (112.62,234.25)	0.773	207.50 (132.20,252.25)	138 (109.25,235.25)	0.274	246 (171.30,299.80)	193.50 (135.65,259)	0.133
Tm1	167.50 (129.87,251.75)	183.95 (137.87,251.75)	0.589	213.25 (85.75,272.50)	164.10 (90,236.50)	0.433	200 (129.12,245)	138.50 (108.25,236.50)	0.297	216 (153.87,303.82)	202.60 (114.20,290.50)	0.355
Tm2	180 (128.25,265.25)	178.75 (120,251.75)	0.819	229 (127.50,328.50)	211 (111.95,302.75)	0.473	211 (127.75,256)	141 (106.75,235)	0.246	238.50 (121,289.52)	227.50 (123.50,282.40)	0.679
Te	181 (129,260)	182.95 (127.82,255)	0.959	230.50 (128.75,336)	216 (110.25,294.50)	0.539	202 (127.82,252.72)	138.45 (106.22,231)	0.308	219.50 (112.87,330)	221.50 (113.75,322.75)	0.756
P value	0.907	0.006		0.850	0.528		0.982	0.294		0.864	0.991	

Data expressed as median (Q1,Q3) or numbers. D, dexmedetomidine; F, fentanyl; n, number of cases; RUL, right upper limb; RLL, right lower limb; LUL, left upper limb; LLL, left lower limb; Ts, baseline latency in the supine position; Tp, latency in the prone position; Tm, latency before any manipulation; Tm1, latency as per the surgeon’s demand; Tm2, latency as per the surgeon’s demand; Te, latency at the end of surgery (Te)

**Table 4. Comparison of Recovery Data, Complications, and Total Propofol Consumption Between the Groups:** 

**Time (min)**	**Group D (n = 30)**	**Group F (n = 30)**	***P* value**
T1	2.04 (1.27)	1.70 (0.81)	0.322
T2	2.08 (1.56)	1.46 (0.67)	0.062
T3	47.55 (7.51) 95% CI (44.863-50.237)	51.10 (8.73) 95% CI (47.976-54.224)	0.046*
Bradycardia	4 (13.33%)	2	0.117
Hypotension	7 (23.33%)	3 (10%)	1.00
Total consumption of propofol (mg)	220 (38) 95% CI (206.402-233.598)	282 (140) 95% CI (231.903-332.097)	0.025*

Data expressed as mean (standard deviation) or numbers, *P value<0.001

D, dexmedetomidine; F, fentanyl; n, number of cases; T1, time for response/eye opening; T2, time to extubation; T3, duration of stay in recovery; CI, confidence interval.
